# Genomic imprinting beyond DNA methylation: a role for maternal histones

**DOI:** 10.1186/s13059-017-1317-9

**Published:** 2017-09-19

**Authors:** Courtney W. Hanna, Gavin Kelsey

**Affiliations:** 1 Epigenetics Programme, Babraham Institute, Cambridge, CB22 3AT UK; 2 Centre for Trophoblast Research, University of Cambridge, Cambridge, CB2 3EG UK

## Abstract

Inheritance of DNA methylation states from gametes determines genomic imprinting in mammals. A new study shows that repressive chromatin in oocytes can also confer imprinting.

## Introduction

Genomic imprinting is an epigenetic phenomenon that allows monoallelic expression of a subset of genes dependent on parental origin and is canonically regulated by DNA methylation. In a recent study, Inoue and colleagues [1] showed that genomic imprinting is also mediated by an oocyte-specific epigenetic mark: the repressive modification of histone tails.

**Fig. 1:**
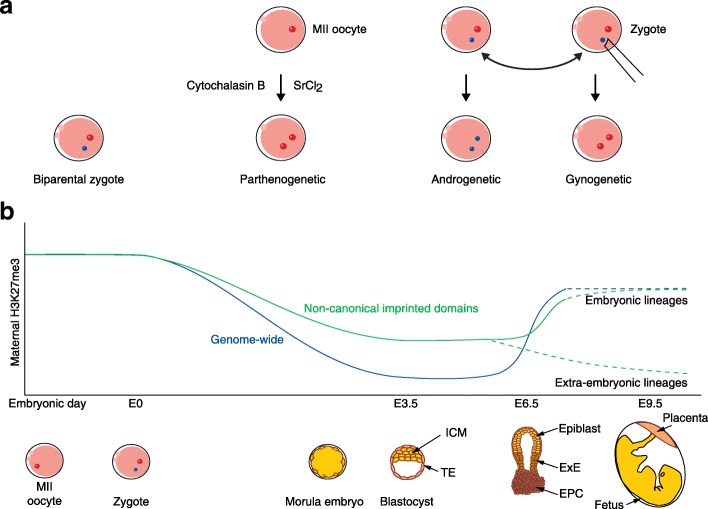
**a** The generation of embryos with only maternal or paternal genetic contributions is a valuable tool in studying genomic imprinting. Parthenogenetic embryos can be generated by treating ovulated metaphase-II (*MII*) oocytes with cytochalasin B to prevent extrusion of the second polar body and artificially activating with strontium chloride (*SrCl*_*2*_). Androgenetic and gynogenetic embryos are created through the micro-manipulation of fertilised zygotes, where the maternal or paternal pronucleus is replaced with the contrasting pronucleus from another zygote. **b** Dynamics of maternally-derived H3K27me3 during embryogenesis. H3K27me3 forms broad domains in the oocyte and, upon fertilisation, maternal H3K27me3 decreases until the formation of the embryonic day 3.5 (*E3.5*) blastocyst [6]. Non-canonical imprinted domains are those that retain relatively more maternal H3K27me3 during this phase of decline, resulting in paternal-specific DNase hypersensitivity sites and gene expression [2]. Post-implantation, there is re-acquisition and re-localisation of H3K27me3 in the embryonic epiblast [6] and correspondingly a loss of non-canonical imprinted domains [2]. Meanwhile, the post-implantation extra-embryonic lineages show a gradual decline in non-canonical imprinting mediated by H3K27me3 [2], although the distribution and localisation of maternal H3K27me3 is currently unknown. *ICM* inner cell mass, *TE* trophectoderm, *ExE* extra-embryonic ectoderm, *EPC* ectoplacental cone

Early embryo manipulation experiments [2], in which embryos were generated with two maternal (parthenogenetic or gynogenetic) or paternal (androgenetic) genomes rather than having biparental contributions (Fig. 1a), showed that the two parental genomes were not functionally equivalent because these manipulated embryos died in early gestation. It was postulated that specific loci in the genome were differentially marked, or ‘imprinted’, between the parental chromosomes. Indeed, it was later shown that monoallelic expression of imprinted genes is predominantly controlled by DNA methylation inherited from the parental germ cells [2]. There are technical limitations in the interrogation of epigenetic states in gametes and early embryos. This means that the question of whether epigenetic modifications other than DNA methylation, such as histone marks, are transmitted from gametes, and whether they are capable of mediating imprinted gene expression, remains outstanding. Utilising gene expression and chromatin accessibility assays in manipulated and hybrid embryos, Inoue and colleagues revealed that a few genomic loci are maternally imprinted because of the inheritance of maternal histone 3 lysine 27 trimethylation (H3K27me3). Defined by the authors as ‘non-canonical’ imprinting, their observations demonstrate a mechanism for the imprinted expression of genes that have previously been reported as independent of DNA methylation [3].

## Intergenerational epigenetic inheritance

Sperm and oocytes exhibit different patterns of chromatin organisation: the sperm DNA is highly methylated and tightly packaged, with protamines replacing most canonical histones, while the oocyte genome has a bimodal methylation pattern, an extensively open chromatin conformation and atypical patterns of histone modifications [4–6]. The paternal genome rapidly loses most of its DNA methylation upon fertilisation, and protamines are replaced by maternal histones [5]. In contrast, the oocyte transmits a considerable amount of epigenetic information to the embryo. Maternal DNA methylation appears to be passively rather than actively erased, and several thousand domains retain some degree of maternally determined DNA methylation by the blastocyst stage [5, 7]. This is consistent with the predominantly maternal contribution to genomic imprinting, with most germline differentially methylated regions (DMRs) being inherited from the oocyte [2, 5]. As well as DNA methylation, recent evidence suggests that maternal histone modifications are preferentially propagated into the embryo [6] and may also form part of the intergenerational epigenetic regulatory landscape.

## Allelic expression in early embryos is conferred by oocyte chromatin

To understand the additional components contributing to intergenerational epigenetic regulation, Inoue et al. [1] undertook a genome-wide characterisation of allelic gene expression and chromatin accessibility in early mouse development, focusing on the one-cell zygote, two-cell embryo and morula-stage embryo. Hybrid embryos were generated from two independent inbred strains; allelic states could thereby be distinguished by aligning sequencing data to genetic variants that corresponded to the maternal and paternal genomes. A limitation of this approach is that even with distantly related mouse strains, not all features can be evaluated allele-specifically. Therefore, to complement this strategy, the authors took advantage of manipulated gynogenetic, parthenogenetic, and androgenetic embryos (Fig. 1a). Within these embryos there is no reliance on strain-specific genetic variants because all data are derived from maternal or paternal alleles, respectively. However, a limitation of using these embryos is that they do not proceed on a normal developmental trajectory [2], so differences between them do not necessarily represent the parental differences observed in normal biparental embryos. By combining the two systems, Inoue et al. presented a powerful approach to study the regulation of genomic imprinting.

The initial evaluation showed that a few hundred loci were monoallelically regulated in the early embryo, with parent-specific DNase hypersensitive sites (DHSs) (representing open chromatin domains) and gene expression. Upon closer investigation, the authors noted that only a subset of the paternal-specific DHSs were associated with DNA methylation in the oocyte. This suggested an alternative mechanism for maintaining a silent maternal allele. Using recently published datasets [6], the authors observed a high level of H3K27me3 in the oocyte and on the maternal allele in embryos at these domains, implicating repressive histone modifications. To test this, they utilised two constructs to modify the endogenous histone modification levels in zygotes by driving overexpression of either an H3K27 or an H3K9 demethylase. The result effectively showed that a subset of paternal-specific DHSs was lost, suggesting that both H3K27me3 and H3K9me3 restrict access to the maternal allele, but at non-overlapping loci.

A series of experiments was carried out in morula-stage embryos to determine the extent to which H3K27me3 continues to silence the maternal allele, thereby mediating paternal-specific DHSs and expression. The authors identified genes associated with paternal DHSs, and then selected those that also showed inherited maternal H3K27me3 methylation. A subset of these genes exhibited paternal allele-biased gene expression. To test whether erasure of maternal H3K27me3 could 'reactivate' gene expression, the H3K27 demethylase KDM6B was injected into parthenogenetic embryos and, notably, both gene expression and DNase sensitivity at several of these genes was increased. This was replicated in hybrid embryos, in which the paternal allele bias of gene expression and chromatin accessibility of these genes was reduced. Together, these findings suggest that maternally inherited H3K27me3 represses gene expression of the maternal allele at several genomic loci. It also raises the question of whether this state is propagated further into development, such as is seen with imprinted DNA methylation.

## Persistence of allelic states occurs preferentially in extra-embryonic tissues

Many genes are specifically imprinted in extra-embryonic tissues [7]. In mouse, embryonic H3K27me3 is known to maintain the imprinting of genes not directly regulated by differential DNA methylation [8]. Therefore, the authors evaluated allelic expression of known non-canonically imprinted genes and the novel candidate genes in blastocysts and extra-embryonic lineages through embryo development up to E9.5. In summary, ~67–80% of assayable candidate genes had paternally biased expression in the blastocyst but, by E9.5, only five non-canonically imprinted genes maintained paternal-specific expression, specifically in the extra-embryonic tissues. These findings provide important evidence of transient imprinting of several loci in the blastocyst-stage embryo that is mediated by the maternal-repressive histone modification H3K27me3. While only a subset of these domains will be propagated into later development, these results nevertheless highlight that genomic imprinting is more pervasive in extra-embryonic tissues.

## Perspectives and open questions

The results of this study suggest there a predominantly transient effect of maternal H3K27me3 at non-canonically imprinted domains during early embryogenesis because these loci are reprogrammed in the embryonic lineages. Furthermore, for the most part, these loci appear to gradually lose alleleic H3K27 trimethylation in extra-embryonic lineages during post-implantation development (Fig. 1b). Importantly, this form of non-canonical imprinting may be functionally significant, because oocyte-specific deletion of EZH2, an H3K27 methyltransferase, severely restricts fetal growth; it is proposed that this is attributed to aberrant placental function [9], and is reminiscent of the growth phenotypes observed with loss of imprinting at several canonically imprinted domains [2].

Interestingly, the transient regulation of gene expression by non-canonical imprinting might also be a mechanism for establishing secondary imprinted DMRs. Secondary imprinted regions are those that show parental-specific DNA methylation, but which are not inherited from the germline. Therefore, the parental bias in methylation between these alleles is established sometime during embryonic development. Duffie et al. [10] characterised the *Gpr1/Zdbf2* locus and established that secondary imprints can occur via the transient monoallelic expression of a promoter-spanning transcript in the early embryo. Thus, transient embryonic paternal gene expression mediated by maternal H3K27me3 could result in the establishment of secondary monoallelic DNA methylation. Investigation of parental DNA methylation within these maternal H3K27me3-regulated transcripts is needed to evaluate this possibility.

A recent study [6] described the dynamics of gamete-determined H3K27 trimethylation in embryogenesis and, strikingly, found that most H3K27me3 was lost by the blastocyst stage (Fig. 1b). While maternal alleles retained more H3K27me3 than paternal alleles, it was preferentially restricted to intergenic GC-poor domains and apparently rapidly reprogrammed at GC-rich promoters. Indeed, Inoue and colleagues [1] also reported the preferentially distal location of allelic DHSs at non-promoter elements. These observations raise two key questions: 1) how H3K27me3 states are reinstated on nascent chromatin in these regions during early embryonic cleavage divisions; and 2) what might govern any specificity towards the non-canonically imprinted domains?

Investigation of the extent to which other maternally inherited histone modifications might regulate gene expression and chromatin accessibility of the embryonic genome has only just begun. The advent of low-input molecular techniques has opened the door for future novel investigations into the establishment of totipotency, the regulation of zygotic genome activation and mechanisms underpinning the first cell lineage specifications in the embryo.

## Abbreviations

DHS: DNase hypersensitive site
H3K27me3: Histone 3 lysine 27 trimethylation
